# Extraction of the electron excess temperature in terahertz quantum cascade lasers from laser characteristics

**DOI:** 10.1515/nanoph-2023-0617

**Published:** 2024-01-10

**Authors:** Nathalie Lander Gower, Shiran Levy, Silvia Piperno, Sadhvikas J. Addamane, John L. Reno, Asaf Albo

**Affiliations:** Faculty of Engineering, Bar-Ilan University, Ramat Gan 5290002, Israel; The Institute of Nanotechnology and Advanced Materials, Bar-Ilan University, Ramat Gan 5290002, Israel; Center for Integrated Nanotechnologies, Sandia National Laboratories, MS 1303, Albuquerque, NM 87185-1303, USA

**Keywords:** terahertz quantum cascade lasers, LO-phonon, electron temperature

## Abstract

We propose a method to extract the upper laser level’s (ULL’s) excess electronic temperature from the analysis of the maximum light output power (*P*_max_) and current dynamic range Δ*J*_d_ = (*J*_max_ − *J*_th_) of terahertz quantum cascade lasers (THz QCLs). We validated this method, both through simulation and experiment, by applying it on THz QCLs supporting a clean three-level system. Detailed knowledge of electronic excess temperatures is of utmost importance in order to achieve high temperature performance of THz QCLs. Our method is simple and can be easily implemented, meaning an extraction of the excess electron temperature can be achieved without intensive experimental effort. This knowledge should pave the way toward improvement of the temperature performance of THz QCLs beyond the state-of-the-art.

Terahertz quantum cascade lasers (THz QCLs) have attracted the attention of the scientific community since their demonstration in 2002 [1]. These lasers provide an effective source for a spectrum that is not accessible by ordinary widespread photonic and electronic devices [2], [3]. Achieving room temperature performance is crucial for enabling the practical application of THz QCLs beyond the confines of the laboratory environment, a goal that remains unattained thus far. Therefore, achieving a high-power portable device has become the primary focus in the field.

The operation of QCLs does not occur in a state of thermal equilibrium, resulting in a potential disparity between the temperature of the electrons and that of the lattice. The presence of an electronic excess temperature plays a critical role in shaping the dynamics of QCLs overall, with a particularly notable impact on their temperature performance. The nonradiative relaxation rate in QCLs strongly depends on the electron temperature, which, at high electron temperatures, significantly affects the gain and threshold current density of THz QCLs [4]. Consequently, optimizing the quantum design of THz QCLs to obtain a strong electron-lattice coupling can lead to improved electrical and optical performance. Furthermore, to achieve a comprehensive understanding of population inversion and optical gain, it is essential to gain knowledge about the actual electron energy distribution, which is determined by the electrons’ temperature [5].

Innovative strategies have been introduced for reducing interfacial thermal resistance in QCLs by investigating the electronic excess temperature [6]. A comprehensive understanding of the lattice temperature, electronic distribution, and electronic temperatures of individual sub-bands is crucial for validating theoretical models and improving the temperature performance of THz QCL active regions [7]. Additionally, the utilization of a combined resonant-tunneling injection and phonon depopulation scheme results in a significant rise in the upper laser level (ULL) temperature, with an approximate increase of ∼100 K at low lattice temperatures (∼10 K) [8].

Previous research has utilized microprobe band-to-band photoluminescence as a means to measure electronic and lattice temperatures and examine sub-band populations [4]–[11]. This technique involves focusing a laser onto the front facet of a THz QCL using an achromatic microscope objective lens. Experimental setup entails a helium-flow micro-cryostat, with temperature monitoring of the heat sink and device copper mount. Careful measures are taken to minimize laser-induced heating, ensuring accurate temperature measurements. Although effective, the employment of microprobe photoluminescence requires extensive experimental and analytical effort. Finding a reliable and simple method to measure the electronic excess temperature should enable a more robust investigation of these devices and eventually lead toward improvement of the temperature performance of THz QCLs.

In this work, we present a method to extract the ULL’s excess electronic temperature from the analysis of the laser’s characteristics. Our method is straightforward and does not require extensive experimental effort. We extract the electron temperature directly from the analysis of the maximum light output power (*P*_max_) and current dynamic range Δ*J*_d_ = (*J*_max_ − *J*_th_) measurements of THz QCLs as a function of temperature.

An effective method for extracting an activation energy (*E*_a_) that points to the temperature sensitive limiting mechanism in THz QCLs by investigating the dependence of measured lasing output power on temperature was previously presented [12]. The *E*_a_ was extracted through an Arrhenius plot of the equation:

(1) $$ae^{\frac{-E_{a}}{kT_{L}}}\approx 1-\frac{P_{\text{out}}\left(T\right)}{{P_{\text{out}}}_{\max}},$$

where $\frac{P_{\text{out}}\left(T\right)}{{P_{\text{out}}}_{\max}}$ is the normalized output power, *a* is a constant, and *T*_L_ is the lattice temperature. This method assumes an electron temperature (*T*_e_) equal to the lattice temperature (*T*_L_), a correct assumption for most devices at lattice temperatures above ∼100 K. In other words, the electron excess temperature (*T*_ex_ = *T*_e_ − *T*_L_) is considered to be equal to 0. In this research, validation was done by studying a set of vertical transition THz QCLs with different emission frequency and the experimental results matched the expectations [12]. The extracted *E*_a_ was of high significance, as it pointed out the temperature sensitive limiting mechanism in the laser. This method was the start of a systematic research that led to the recent recorded maximum operating temperature (*T*_max_) of ∼250 K [13] and ∼261 K [14].

To summarize the use of this method, a brief review of this systematic research is presented below. The extracted value of *E*_a_ for standard vertical-transition THz QCLs was of ∼20 meV, which matched the thermally activated longitudinal optical (LO) phonon scattering from the ULL to the lower lasing level (LLL) [12]. To overcome this limitation, highly diagonal structures were designed, effectively reducing the impact of thermally activated LO phonon scattering [15], [16]. However, in highly diagonal THz QCLs, the extracted *E*_a_ was of ∼80 meV, pointing to another limiting mechanism. The main mechanisms observed to restrict temperature performance in these structures was the thermally activated leakage into the continuum and the thermal leakage of charged carriers into excited bound states. Leakage into the continuum was particularly significant when using barriers with only 15 % Al [16], [17]. In devices with higher potential barriers containing 30 % Al, leakage into the continuum was reduced, but thermally activated leakage into excited bound states was still observed [18], [19]. By combining high barriers with thin wells, it has been possible to elevate the energies of excited bound and continuum states and suppress these leakage pathways [19]–[21]. Carefully engineered devices have exhibited clear negative differential resistance (NDR) behavior in current–voltage (I–V) curves up to room temperature, indicating electron transport exclusively within the laser’s active sub-bands and the suppression of thermally activated leakage paths [19]–[23]. In these new highly diagonal devices, the extracted *E*_a_ was again ∼20 meV, pointing at the LO-phonon scattering from the ULL to the LLL as the main limiting mechanism, meaning the previous observed leakage into the continuum and into excited bound states mechanisms were diminished. This same strategy led to the design of a two-well structure with suppressed thermally activated leakage channels, where all transport occurs only within the active laser states [20], i.e., clean three-level system. This scheme paved the way for the latest THz QCLs advancements with a *T*_max_ of ∼250 K [13] and ∼261 K [14]. Recognizing the temperature sensitive limiting mechanism in THz QCLs by extracting the *E*_a_ proved to be a reliable method on all the aforementioned devices.

However, some devices had characteristics that were harder to analyze, such was the case of devices with extracted *E*_a_ values much lower than ∼20 meV, which are not physical. The main issue is that the method assumes an electron temperature equal to the lattice temperature, and in these devices, there appeared to be a significant excess electron temperature (*T*_ex_), which is why the method of extracting *E*_a_ did not work. This drove us to develop a novel approach that accounts for situations where the electron temperature (*T*_e_) exceeds the lattice temperature (*T*_L_). We assume a known *E*_a_ and focus on the extraction of *T*_ex_ through the deviation of the calculated *E*_a_ from the expected one, as will be explained.

Our new approach is based on the method previously presented [12], but instead of the lattice temperature, we considered the electron temperature. Then the new equation looks as follows:

(2) $$ae^{\frac{-E_{a}}{kT_{e}}}\approx 1-\frac{P_{\text{out}}\left(T\right)}{{P_{\text{out}}}_{\max}}.$$

In order to explore what happens when the electron temperature is higher than the lattice temperature (*T*_e_ > *T*_L_), we conducted calculations on a clean three-level system, which represents the typical structure observed in recent advancements of THz QCLs [13], [14], [19], [20], [21] and is the simplest scheme to analyze. In a clean three-level system, the main limiting mechanism is usually the thermally activated LO-phonon scattering from the ULL to the LLL and the relevant *E*_a_ to this process can be calculated from the laser’s spectrum measurements, i.e., *E*_a_ = *E*_LO_ − *hν*. A schematic of a clean three-level THz QCL model is shown in Figure 1a. In this model, the only thermally activated mechanism is the LO-phonon scattering from the ULL to the LLL, making it the simplest model to examine. Thermal backfilling could be another potential thermally activated mechanism, but its activation energy is much higher; hence, it is less probable to be the main limiting mechanism. Although it is essential to acknowledge that thermal backfilling can indeed be a limiting factor in some THz QCL designs [24], [25], [26], former research also indicates that this is not the mechanism limiting the temperature performance in most cases [27]. Moreover, in clean three-level systems, the separation between LLL and injector is usually greater than the LO-phonon energy (around ∼55 meV), due to the thinner wells, making thermal backfilling even less likely to affect the temperature performance.

**Figure 1:**
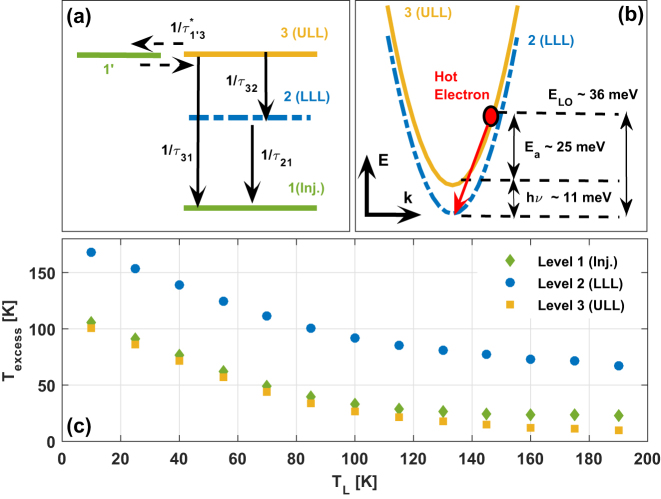
(a) Schematic of a clean three-level THz QCL model. (b) Illustration of the thermally activated intersub-band LO-phonon scattering process from the ULL to the LLL. (c) Calculated sub-band excess temperatures for a typical three-level THz QCL design as a function of lattice temperature.

In Figure 1b, we show an illustration of the thermally activated intersub-band LO-phonon scattering process from the ULL to the LLL. As can be seen, the hot electron relaxes through LO-phonon scattering, where the sum of the photon energy and the *E*_a_ being equal to the LO-phonon energy. We chose a relatively large *E*_a_ (∼25 meV) that enables us to effectively illustrate the plots and enhance the clarity of the visualization.

A calculation of the sub-band excess temperatures for a typical three-level THz QCL design as a function of lattice temperature is presented in Figure 1c. The electron excess temperatures in Figure 1c were calculated using a rate equation model with energy balanced conditions. The specific data presented in the figure were obtained from a classical two-well design, but it’s important to note that the results are consistent with those from other clean *n*-level systems. As can be seen, the excess temperature of the electrons in levels 1 (injector) and 3 (ULL) starts at around ∼100 K at low lattice temperatures and drops with increasing lattice temperature, meaning the electrons cool down. Above a lattice temperature of ∼100 K, the excess temperature remains about constant. Based on this, we can assume a constant excess temperature of the ULL for lattice temperature values above ∼100 K. The electronic temperature is similar in the ULL and injector, and we will assume this for further calculations. It can be noticed that the temperature of the LLL electrons is even higher; however, in the most recent devices, i.e., clean three-level system devices, the performance is not affected by the temperature of the electrons in the LLL, as there are no leakages to continuum or excited states from the LLL unlike former designs [16], [28]. This is also verified by the results of the simulations where no sensitivity to the LLL temperatures was observed.

We can observe in Figure 2a the plots for the normalized photon flux as function of the lattice temperature (*T*_L_) in linear scale. The calculations were performed using a rate equation model as described in Ref. [12] and references therein. We consider the normalized output power $\frac{P_{\text{out}}\left(T\right)}{{P_{\text{out}}}_{\max}}$ to be equal to the normalized flux $\frac{S\left(T\right)}{S_{\max}}$ as described in Ref. [12]. The calculations were conducted for a lasing frequency of 11 meV (2.66 THz), *E*_a_ = 25 meV, and an ULL’s excess temperature (*T*_ex_) of 0 K, 20 K, 40 K, and 60 K. From Figure 2a, we can derive *T*_max_ for each plot by identifying the point at which the normalized flux reaches zero. In other words, *T*_max_ is the temperature at which the normalized flux is no longer discernible. There is a high sensitivity of the normalized flux (output power) to the ULL’s excess electron temperature, as shown by the drop of *T*_max_ with increasing *T*_ex_, meaning the temperature performance of the lasers decreases drastically as *T*_ex_ rises.

**Figure 2:**
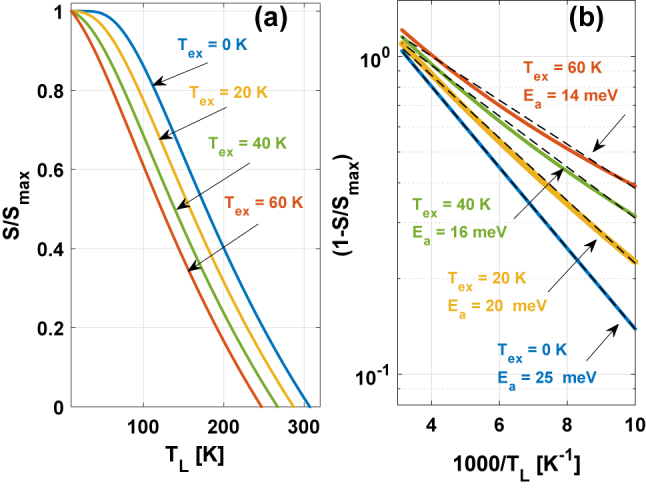
(a) Normalized photon flux (output power) as function of the temperature in linear scale. (b) A semi-logarithmic plot of $\left(1-\frac{S\left(T\right)}{S_{\max}}\right)$ , where *S* is the calculated photon flux according to equation (1) in [12] and $\frac{P_{\text{out}}\left(T\right)}{{P_{\text{out}}}_{\max}}\approx \frac{S\left(T\right)}{S_{\max}}$ , with the excess temperature inputs for the ULL and the fitted activation energy values. The calculations were performed using a rate equation model as described in Ref. [12] and references therein.

A semi-logarithmic plot of $\left(1-\frac{S\left(T\right)}{S_{\max}}\right)$ is presented in Figure 2b. Then, considering Equation (2), the effective *E*_a_ can be derived from the slope of $\ln\left(1-\frac{S\left(T\right)}{S_{\max}}\right)$ versus inverse lattice temperature (*T*_L_). When *T*_ex_ = 0 *K*, then *E*_a_ = 25 meV as expected. As can be seen, the slope of the curve is lower as *T*_ex_ increases, indicating an *E*_a_ value, which is physically incorrect and a sign of how the excess temperature of the electrons in the ULL affects this value. Assuming we know which mechanism is the one limiting the laser’s performance, we can know the physical value of *E*_a_ and then we can extract from the change of the slope in these plots the *T*_ex_ value. As explained before, in a clean three-level system, the value of *E*_a_ is a known value and can be predicted from the lasing frequency, according to *E*_a_ = *E*_LO_ − *hν*. Consequently, the change in the slope serves as our indicator of the *T*_ex_ value. In summary, we have devised a method for the extraction of *T*_ex_, and its validity has been confirmed through computational analysis.

In order to experimentally validate our method, we studied a split-well direct-phonon (SWDP) GaAs/Al_0.3_Ga_0.7_As THz QCL supporting a clean three-level system [21], [29] (Figure 3a). Characterization of the laser was performed under pulsed operation corresponding to an extremely low duty cycle to avoid device heating; hence, the excess electron temperature measured is a result only of the electron dynamics. The pulse width was 400 ns at 500 Hz, equating to a 0.02 % duty cycle. The structure of the devices consists of three active laser sub-bands in each module; all other levels are considered parasitic. A very fast depopulation rate of the LLL is reached, by means of resonant LO-phonon scattering only. Carrier leakage channels are reduced by means of a thin intrawell barrier that pushes excited states to higher energies. The ULL, LLL, and the injector level are aligned in the direct phonon scattering scheme. 

**Figure 3:**
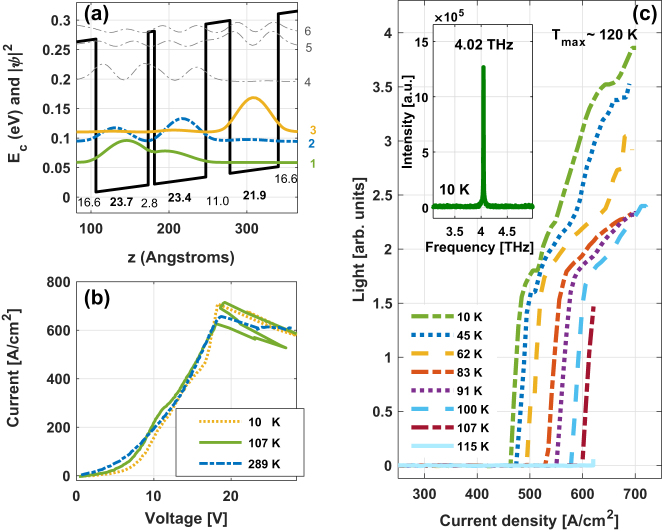
(a) Band diagram of one period of a SWDP design supporting clean three-level system THz QCL with 30 % Al in all barriers. (b) Current curves as a function of voltage at low, around maximum operating, and room temperatures. (c) Pulsed light–current and spectrum (inset) measurements of the SWDP design. The measured maximum operating (lasing) temperature is ∼120 K and emission frequency ∼4.02 THz, as indicated. Ref. [29].

The current versus voltage (I–V) curves in Figure 3b demonstrate that the device has negative differential resistance (NDR) all the way up to room temperature. This indicates an effective isolation of the three active laser states from the excited and continuum states, i.e., a clean three laser-level system was obtained in this device. We observe fluctuations in the I–V curves close to *T*_max_, indicating output power instability [29]. The occurrence of fluctuations in the I–V curves is correlated with the fast deterioration of the laser’s intensity. The L–I curves of the device are presented in Figure 3c. The measured maximum operating (lasing) temperature is ∼120 K and emission frequency ∼4.02 THz, as indicated.

Since the current dynamic range is proportional to the maximum output power, an activation energy can be also extracted from this laser characteristic. Nonetheless, the assumption here is that *J*_th_ approximates the nonlasing current *J*_nl_ (the current that will be measured on a nonlasing device at the biasing condition of *J*_max_), as described by the equation Δ*J*_d_ = (*J*_max_ − *J*_th_) ≈ (*J*_max_ − *J*_nl_) ∝ *P*_out_. Consequently, the fit of the current dynamic range data can be affected by underestimation of the electron excess temperature, as the electron temperature may increase at the nonlasing maximum current biasing conditions with respect to the threshold biasing conditions [4], [10], [11]. In this device, which supports a clean n-level system with no parallel leakage affecting *J*_max_, this value was directly obtained from the I–V curves [21]. However, it’s important to note that in structures that don’t support clean *n*-level systems, *J*_max_ could also be derived from L–I curves, as, in such cases, the presence of parallel leakage might affect the I–V curves.

In our experimental results (Figure 4), we can observe that given the analysis of the *P*_out_ data, we extracted an activation energy value of ∼5 meV, a different value from the one extracted from the spectrum measurement (Figure 3c) for the thermally activated LO-phonon scattering mechanism (∼20 meV). Additionally, a sharp drop can be seen at the beginning of the *P*_out_ curve (higher temperatures), pointing also to an output power instability. On the other hand, from the measured current dynamic range of the device, we extracted an experimental activation energy of ∼19 meV from the high temperatures side of the data, which matches the predicted *E*_a_, this is due to the underestimation of *T*_ex_. Then, the excess electronic temperature can be easily extracted from the comparison of the laser’s maximum output power data and the current dynamic range data as previously explained. At lower temperatures, the current dynamic range curve deviates from this slope (green circles, Figure 4), indicating the presence of nonzero electron excess temperature. We can also observe that the slope of the dynamic range curve (green circles, Figure 4) at the low temperatures side of the data is similar to the slope of the *P*_max_ curve (red circles, Figure 4).

**Figure 4:**
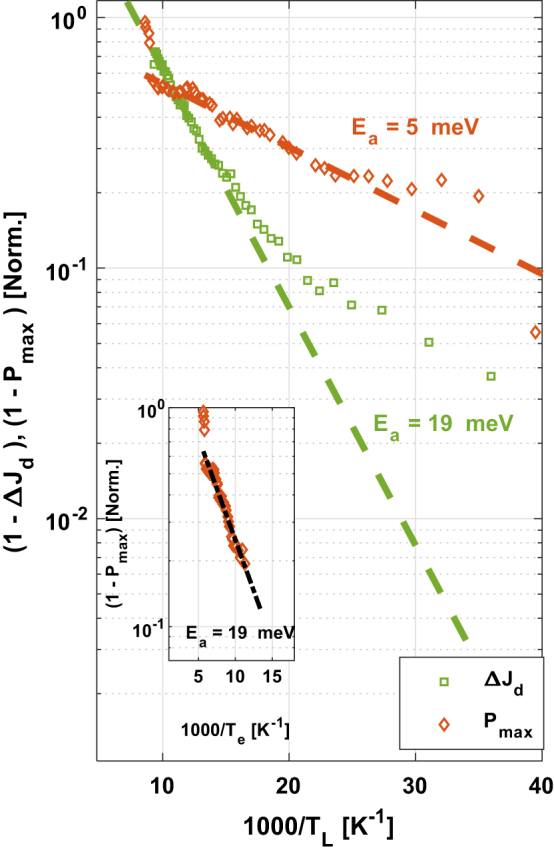
Activation energy extracted from the current dynamic range Δ*J*_d_ = (*J*_max_ − *J*_th_) (green squares) and the laser’s maximum power output (*P*_max_) (brown diamonds). The quantities in the *y*-axis are $\left(1-\frac{\Delta J_{d}\left(T\right)}{\Delta {J_{d}}_{\max}}\right)$ and $\left(1-\frac{P_{\text{out}}\left(T\right)}{{P_{\text{out}}}_{\max}}\right)$ respectively presented in logarithmic scale. An Arrhenius plot for $\left(1-\frac{P_{\text{out}}\left(T\right)}{{P_{\text{out}}}_{\max}}\right)$ is presented in the inset, this time as a function of the electron temperature *T*_e_, including a characteristic excess temperature of ∼60 K, resulting in an activation barrier of ∼19 meV for the laser’s maximum power output (*P*_max_) (brown diamonds) data, similarly to the current dynamic range Δ*J*_d_ = (*J*_max_ − *J*_th_) (green squares in main Figure) data with zero excess electron temperature.

The low activation energy that we observed in the *P*_max_ curve (Figure 4) is not the real physical activation energy since the electrons at the ULL are much hotter than the lattice. Inclusion of a characteristic excess temperature of *T*_ex_ ∼ 60 K in an Arrhenius plot presented as a function of the total electron temperature rather than the lattice temperature (Figure 4 inset) would result in an activation barrier of ∼19 meV, which is like that extracted from the high temperature side of the current dynamic range data and also from the measured spectrum [12], [29]. Upon closer examination of the graph, it can be observed that the data points in Figure 4 inset do not deviate from the slope at lower temperatures. We consider that the lower slope observed for the *P*_out_ data analysis allows us to probe a characteristic excess electron temperature through a comparison with the slope of the current dynamic range at high temperatures that underestimates the excess electron temperature and with the activation energy value extracted from the measured spectrum, i.e., a characteristic excess temperature of *T*_ex_ ∼ 60 K. As indicated from the temperature span of the *P*_out_ curve, this excess temperature remains up to temperatures of ∼120 K unlike for formerly reported THz QCLs where electrons already cooled down to the lattice temperature at these temperatures [12], [19], [21]. We attribute the fact that the electrons remain hotter than the lattice at temperatures above ∼100 K to the combination of the very low oscillator strength (*f* ∼ 0.2) and the full suppression of leakage paths in our clean three-level device.

As we understood the full suppression of leakage paths could be the cause for the presence of *T*_ex_ at high lattice temperatures, we realized another clean three-level design. However, in this scheme the barriers are lower, making the structure more lenient toward hot electron leakages, thus, allowing hot electrons to cool down more effectively. This structure is presented in Figure 5a. This is also a SWDP design but with mixed potential barriers, GaAs/Al_0.55_Ga_0.45_As injector barrier and GaAs/Al_0.15_Ga_0.85_As radiative and intrawell barriers [21]. The current versus voltage (I–V) curves in Figure 5b demonstrate that this device also has negative differential resistance (NDR) all the way up to room temperature. This indicates an effective isolation of the three active laser states from the excited and continuum states, i.e., a clean three laser-level system was obtained in this device too. Additionally, the I–V curves close to *T*_max_ are smoother in comparison to the curves observed for the former device in Figure 3b, pointing to a more stable lasing [29]. However, a slight change in the slope of the curves can still be observed, this is pointed by an arrow in Figure 5b. The L–I curves of the device are presented in Figure 5c. The measured maximum operating (lasing) temperature is ∼170 K and emission frequency ∼2.43 THz, as indicated.

**Figure 5:**
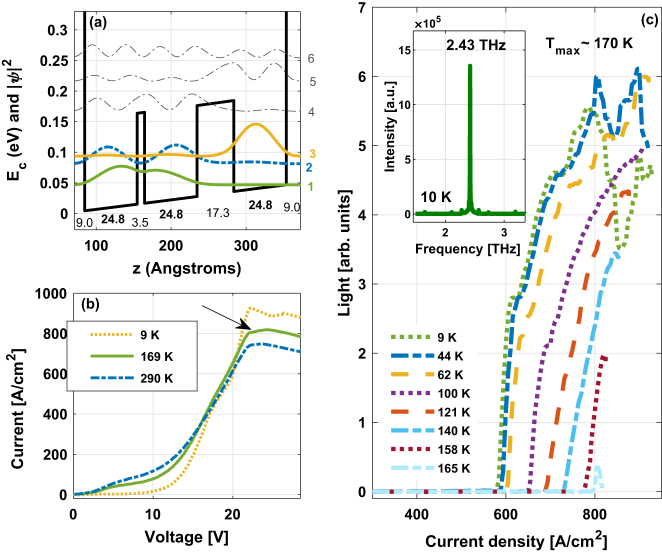
(a) Band diagram of one period of a SWDP design supporting clean three-level system with mixed barriers: Al_0.55_Ga_0.45_As injection barrier (nominally pure AlAs barrier) and Al_0.15_Ga_0.85_As radiative and intrawell barriers. (b) Current curves as a function of voltage at low, around maximum operating, and room temperatures. An arrow points to a slight change in the curves, assumingly from residual fluctuations. (c) Pulsed light–current and spectrum (inset) measurements of the SWDP design with mixed barriers. The measured maximum operating (lasing) temperature is ∼170 K and emission frequency ∼2.43 THz, as indicated. Ref. [21].

As done for the first design, we present the analysis of the *P*_out_ data in Figure 6. Given the measured current dynamic range of the device, we extracted an experimental activation energy of ∼26 meV from the high temperatures side of the data. A similar value is extracted from the spectrum measurement (Figure 5c), for the thermally activated LO-phonon scattering mechanism. Unlike the design before, here at lower temperatures the current dynamic range curve does not deviate as much from its initial slope (green circles, Figure 6). Moreover, the result observed from the analysis of the *P*_out_ data also matches this result of ∼26 meV, indicating the electron temperature in this scheme is similar to the lattice temperature. In other words, the graphs result in a slope that closely aligns with the slope predicted from the frequency measurements (according to *E*_a_ = *E*_LO_ − *hν*), leading us to the conclusion that *T*_ex_ = 0. We can still observe a sharper drop at the beginning of the *P*_out_ curve (higher temperatures), also pointing to some residual output power instability. The measured maximum operating (lasing) temperature in this design was ∼170 K, indicating an enhancement over the former design was the value of *T*_max_ was ∼ 120 K, meaning the temperature performance was improved by ∼50 K. This serves as a notable demonstration of the substantial enhancement we can achieve in *T*_max_ by effectively reducing *T*_ex_, through the implementation of our innovative approach.

**Figure 6:**
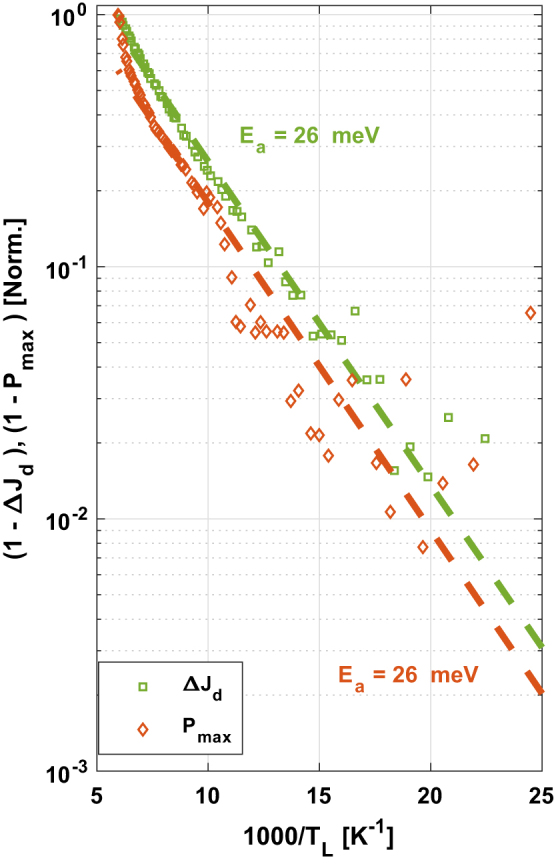
Activation energy extracted from the current dynamic range Δ*J*_d_ = (*J*_max_ − *J*_th_) (green squares) and the laser’s maximum power output (*P*_max_) (brown diamonds). The quantities in the *y*-axis are $\left(1-\frac{\Delta J_{d}\left(T\right)}{\Delta {J_{d}}_{\max}}\right)$ and $\left(1-\frac{P_{\text{out}}\left(T\right)}{{P_{\text{out}}}_{\max}}\right)$ respectively presented in logarithmic scale.

To summarize, we developed a new and straightforward method to extract the *T*_ex_ in THz QCLs. We corroborated our method through simulations and subsequently verified it experimentally. The effectiveness of this method has not only been validated through our case study but also proved to be a useful tool toward the enhancement of *T*_max_, as demonstrated by the encouraging results of the second scheme proposed. The results obtained from the implementation of this method on proposing this scheme emphasize its utility as a valuable tool in maximizing *T*_max_.

It is critical to emphasize that modern THz QCLs primarily rely on clean 3-level systems. This emphasizes the need of knowing and reducing excess electron temperature (*T*_ex_) in these systems’ ULL. *T*_ex_ in the ULL is especially identified as a potential problem in these advanced THz QCLs by our research. This phenomenon can be attributed, in part, to the lack of effective leakages that cool down the electrons within these devices. In this paper, we also propose a potential solution, which is designing new devices with a more lenient structure toward leakages through the incorporation of mixed potential barriers. Our model provides a thorough framework for analyzing and optimizing THz QCLs, making it a useful tool in this regard. We may obtain vital insights into the properties of these high-performing devices by researching their characteristics. This approach can be implemented on devices that have already established *T*_max_ records, potentially improving their temperature performance and propelling THz QCL technology to new heights.

Future work would include the application of our *T*_ex_ extraction method in these cases to other designs. The excess temperature of the electrons and its dependance on the lattice temperature could potentially be extracted solely from the maximum output power data analysis for other THz QCLs. However, comparing both, the current dynamic range data, and maximum output power analysis, should provide us with a more detailed analysis of the electron excess temperature. Our method is simple and can be easily implemented, meaning an extraction of the excess electron temperature can be achieved without intensive experimental and analytical effort as done in Ref. [3–11]. This knowledge should pave the way toward improvement of the temperature performance beyond the state-of-the-art.
